# Variable frequency of *LRRK2* variants in the Latin American research consortium on the genetics of Parkinson’s disease (LARGE-PD), a case of ancestry

**DOI:** 10.1038/s41531-017-0020-6

**Published:** 2017-06-02

**Authors:** Mario Cornejo-Olivas, Luis Torres, Mario R. Velit-Salazar, Miguel Inca-Martinez, Pilar Mazzetti, Carlos Cosentino, Federico Micheli, Claudia Perandones, Elena Dieguez, Victor Raggio, Vitor Tumas, Vanderci Borges, Henrique B. Ferraz, Carlos R. M. Rieder, Artur Shumacher-Schuh, Carlos Velez-Pardo, Marlene Jimenez-Del-Rio, Francisco Lopera, Jorge Chang-Castello, Brennie Andreé-Munoz, Sarah Waldherr, Dora Yearout, Cyrus P. Zabetian, Ignacio F. Mata

**Affiliations:** 1 Neurogenetics Research Center, Instituto Nacional de Ciencias Neurologicas, Lima, Peru; 2 Northern Pacific Global Health Research Training Consortium, Bethesda, MD USA; 3 Movement Disorders Unit, Instituto Nacional de Ciencias Neurologicas, Lima, Peru; 4 Universidad Nacional Mayor de San Marcos, Lima, Peru; 5 Universidad Peruana Cayetano Heredia, Lima, Peru; 6 Hospital de Clínicas José de San Martín, Universidad de Buenos Aires, Buenos Aires, Argentina; 7 Neurology Institute, Universidad de la Republica, Montevideo, Uruguay; 8 Department of Genetics, Facultad de Medicina, Universidad de la Republica, Montevideo, Uruguay; 9 Ribeirão Preto Medical School, Universidade de São Paulo, Ribeirão Preto, Brazil; 10 Movement Disorders Unit, Department of Neurology and Neurosurgery, Universidade Federal de São Paulo, São Paulo, SP Brazil; 11 Hospital de Clínicas de Porto Alegre, Porto Alegre, Brazil; 12 Neruroscience Research Group, Medical Research Institute, Universidad de Antioquia, Medellin, Colombia; 13 Department of Genetics, School of Medicine, Universidad de Guayaquil, Hospital Luis Vernaza, Guayaquil, Ecuador; 14 Service of Neurology, Hospital Luis Vernaza, Guayaquil, Ecuador; 15 Veterans Affairs Puget Sound Health Care System, University of Washington, Seattle, WA USA; 16 Department of Neurology, University of Washington, Seattle, WA USA

## Abstract

Mutations in *Leucine Repeat Rich Kinase 2 (LRRK2)*, primarily located in codons G2019 and R1441, represent the most common genetic cause of Parkinson’s disease in European-derived populations. However, little is known about the frequency of these mutations in Latin American populations. In addition, a prior study suggested that a *LRRK2* polymorphism (p.Q1111H) specific to Latino and Amerindian populations might be a risk factor for Parkinson’s disease, but this finding requires replication. We screened 1734 Parkinson’s disease patients and 1097 controls enrolled in the Latin American Research Consortium on the Genetics of Parkinson’s disease (LARGE-PD), which includes sites in Argentina, Brazil, Colombia, Ecuador, Peru, and Uruguay. Genotypes were determined by TaqMan assay (p.G2019S and p.Q1111H) or by sequencing of exon 31 (p.R1441C/G/H/S). Admixture proportion was determined using a panel of 29 ancestry informative markers. We identified a total of 29 Parkinson’s disease patients (1.7%) who carried p.G2019S and the frequency ranged from 0.2% in Peru to 4.2% in Uruguay. Only two Parkinson’s disease patients carried p.R1441G and one patient carried p.R1441C. There was no significant difference in the frequency of p.Q1111H in patients (3.8%) compared to controls (3.1%; OR 1.02, *p* = 0.873). The frequency of *LRRK2*-p.G2019S varied greatly between different Latin American countries and was directly correlated with the amount of European ancestry observed. p.R1441G is rare in Latin America despite the large genetic contribution made by settlers from Spain, where the mutation is relatively common.

## Genetic risk: Uncovering ethnic-specific mutations

A new study reveals the frequency of *Leucine-Rich Repeat Kinase 2* (*LRRK2*) mutations associated with Parkinson’s disease (PD) in Latin Americans. Ignacio F. Mata at the University of Washington and the VA Puget Sound Health Care System, Seattle, USA, and colleagues from six South American countries have screened the largest cohort of Latino PD patients ever assembled (1739) and 1104 healthy controls for *LRRK2* mutations that are known to cause PD in European-derived populations. They found that the p.G2019S missense mutation was the most common, although its frequency varied greatly between countries and was directly correlated with European ancestry. In contrast, the p.R1441G mutation which is common in Spain is rare in Latin America. Further analyses of this cohort will help to further characterize the genetic profile of PD patients in Latin America and contribute to the development of personalized medicines.

## Introduction

Mutations in *Leucine Repeat Rich Kinase 2* (*LRRK2*) represent the most frequent genetic cause of Parkinson’s disease (PD); there is consistent evidence that at least eight missense variants (p.N1437H, p.R1441C, p.R1441G, p.R1441H, p.R1441S, p.Y1699C, p.G2019S, and p.I2020T) are pathogenic while more than fifty remain of undetermined significance.^1, 2^ p.G2019S is the most frequent pathogenic *LRRK2* variant in European-derived, Ashkenazi Jewish, and North African populations, however frequencies range from 0 to 42% worldwide depending on the population.^3^ While most carriers share a common founder,^4, 5^ a small group of patients in Europe and Japan share two different haplotypes, suggesting at least three different mutation events.^6, 7^ Another mutation, p.R1441G, is almost exclusively restricted to Northern Spain and is thought to have originated in the “Basque” region during the seventh century.^8, 9^ Only four patients have been reported to carry this variant outside of Spain: one in Mexico,^10^ one in the US,^11^ one in Uruguay^12^ and more recently one in Japan.^13^ Three other less common pathogenic variants (p.R1441C, p.R1441H, and p.R1441S) are known to occur within the same codon.^4, 14, 15^ Thus p.G2019S, together with variants in the R1441 codon, represent the two largest mutational hotspots in the gene with highly variable frequencies depending on geographic location and ethnic background. There are other variants in *LRRK2* that are population specific and associated with PD risk such as the p.G2385R and p.R1628P single nucleotide polymorphisms (SNPs) in Asians.^16^

Little is known about the frequency of these or other *LRRK2* variants in Latin American countries. In prior studies of small cohorts from South America our group and others have shown that the frequency of p.G2019S and p.R1441G varies substantially across different countries.^3, 12, 17, 18^ In a small pilot study we also observed that the *LRRK2-*p.Q1111H SNP, which is common in some Latin American populations, occurred at an increased frequency in PD patients, though the difference was not quite significant.^19^ Thus, whether this variant represents a PD risk factor in Latino populations remains unclear.

In this study we sought to further elucidate the frequency of the *LRRK2*-p.R1441G/C/H/S and p.G2019S mutations and the influence of p.Q1111H on disease risk in the largest cohort of Latin American PD patients ever examined.

## Results

**Fig. 1:**
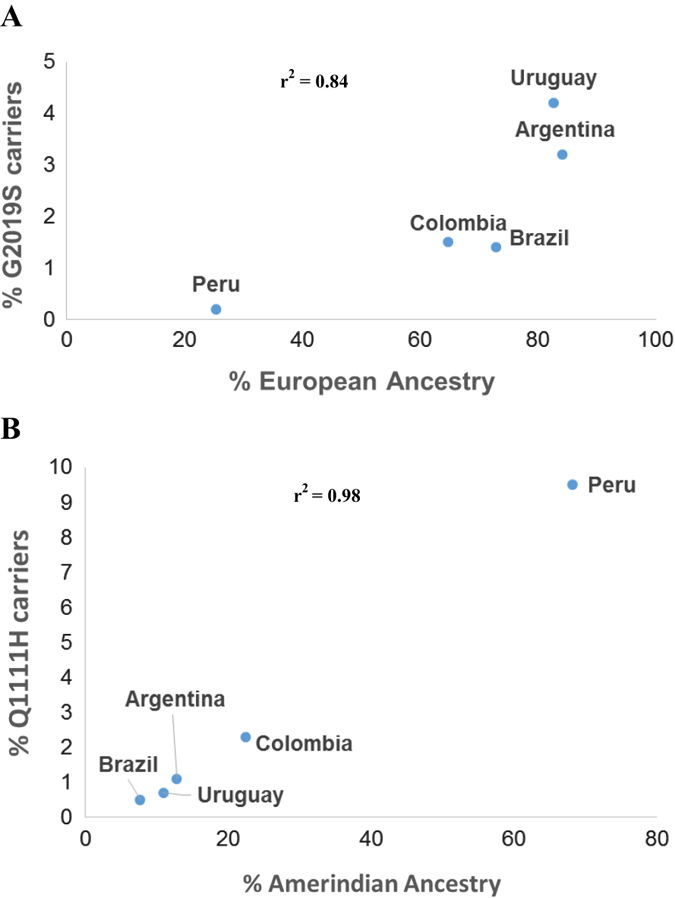
Correlation between frequency of **a** p.G2019S and **b** p.Q1111H carriers and the proportion of European or Amerindian admixture in the cohort from each site. Correlation is shown in each of the figures as the coefficient of determination (*r*^2^)

We identified a total of 29 patients who carried p.G2019S, including one homozygote (from Argentina). p.G2019S frequency varied substantially between sites, and was strongly correlated with the proportion of European admixture observed in representative samples from the corresponding site (Table 1; Fig. 1a). All but two of the carriers had an age at onset over 40 years old (mean 54.7, range 37–72). All carriers reported at least one European ancestor, and only five reported a family history of PD. No sex differences were observed as exactly 50% were females. Nine unaffected relatives of four of the carriers were also found to harbor p.G2019S. Their ages ranged from 19 to 55 years old. Five healthy controls (age at recruitment 39–77) were also found to carry p.G2019S.

**Table 1:** Summary characteristics of the study population

	Argentina		Brazil		Colombia		Peru		Uruguay		Ecuador	
	Cases	Controls	Cases	Controls	Cases	Controls	Cases	Controls	Cases	Controls	Cases	Controls
N	188	0	433	352	197	184	543	255	288	306	85	0
AAD	60.5 ± 13.0	NA	63.5 ± 12.0	58.8 ± 12.2	66.2 ± 13.2	53.1 ± 14.1	62.3 ± 11.9	58.2 ± 13.5	63.3 ± 12.4	60.9 ± 12.2	67.6 ± 10.5	NA
AAO	53.8 ± 12.9	NA	55.6 ± 13.1	NA	51.2 ± 16.5	NA	57.3 ± 13.0	NA	56.6 ± 13.1	NA	62.3 ± 11.8	NA
% Males	55.90%	NA	56.7%	24.8%	47.1%	46.6%	54.5%	29.0%	50.3%	35.5%	56.0%	NA
% European^a^	84.1%		72.9%		64.7%		25.3%		82.7%		NA	
% Amerindian^a^	12.8%		7.7%		22.4%		68.1%		10.9%		NA	
G2019S (%)	6^b^/185 (3.2%)	NA	6/423 (1.4%)	0/289	3/196 (1.5%)	1/184 (0.5%)	1^c^/542 (0.2%)	1/228 (0.4%)	12^c^/283 (4.2%)	3/275 (1.1%)	1/85 (1.2%)	NA
R1441G (%)	0/181	NA	0/420	0/241	0/197	0/184	1/534 (0.2%)	0/247	1^c^/270 (0.4%)	0/265	0/85	NA
R1441C (%)	0/181	NA	1/420 (0.2%)	0/241	0/197	0/184	0/534	0/247	0/270	0/265	0/85	NA

^a^ Estimated average ancestry calculated using 17–50 randomly selected individuals from each site using 29 ancestry informative markers and unsupervised learning algorithm STRUCTURE

^b^ Includes one homozygote

^c^ 6 p.G2019S carriers and 1 p.R1441G from Peru and Uruguay were described in a previous publication [12]

Genotyping of rs28903073 showed that all 34 p.G2019S carriers (29 patients and 5 controls) carried at least one copy of the minor allele (A), thus suggesting that they all share the most common haplotype reported among individuals with p.G2019S in Europe and North America.^4^

**Fig. 2:**
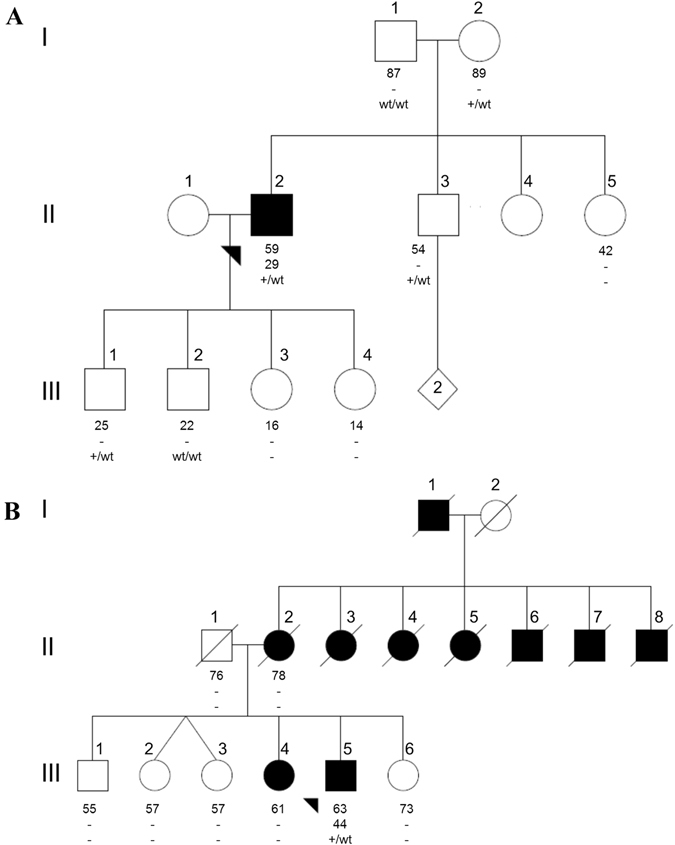
Pedigree diagram of the **a** Peruvian p.R1441G and **b** Brazilian p.R1441C families. Individuals affected with Parkinson’s disease are represented with *black* symbols, unaffected individuals with *open* symbols. Age at last clinical evaluation (or age at death if individual is deceased) is indicated immediately below each symbol, followed by age at onset; +, mutation carrier; wt, wild-type

We also identified three patients who were heterozygous for a pathogenic mutation in codon 1441. Two carried p.R1441G (from Uruguay and Peru) and one carried p.R1441C. All three had an age at onset ≤50 years old. Only the p.R1441C carrier reported a family history of PD (Fig. 2b), despite the fact that mutations in this codon are highly penetrant.^20, 21^ We screened five additional unaffected family members of the Peruvian patient with p.R1441G, including both of his parents who are still alive in their late 80s, and identified three more carriers (Fig. 2a). All carriers from this family shared the same haplotype that has been reported among p.R1441G carriers in the “Basque” region (Supplementary Table 1). We did not find other pathogenic variants in exon 31 for any patients or controls.

We also screened our cohort for the p.Q1111H polymorphism and found 118 patients (10 homozygotes) and 58 healthy controls (3 homozygotes) who carried this variant. The allele frequency was highly variable between sites and was highly correlated with the proportion of Amerindian admixture observed at each site (Fig. 1b). However, there was no significant difference in p.Q1111H frequency between cases and controls in the combined sample (or by site) after adjusting for age, sex and site (Table 2).

## Discussion

Our findings indicate that the frequencies of the *LRRK2* p.G2019S and p.R1441G/C mutations vary widely across countries in Latin America, and are strongly linked to the amount of European admixture existent in each country. We observed a high p.G2019S frequency in Argentina (3.2%) and Uruguay (4.2%), the two countries with the highest proportion of European admixture (>85%). In contrast, the prevalence of the mutation was lower in Peru (0.2%), where European admixture is only about 25%. Frequencies in Ecuador (1.2%), Colombia (1.5%) and Brazil (1.4%) are similar to those observed in other studies in Latin America and in the US.^3^ This is the first time that the p.G2019S has been reported in Ecuador.

Our data indicate that all of the individuals in our sample carrying p.G2019S share the haplotype most commonly reported among carriers of this mutation in European-derived, Ashkenazi Jewish, and North African populations. These individuals are all believed to share a common founder who lived more than 2000 years ago in the Middle East.^4^

In contrast to the widespread distribution of p.G2019S, mutations in codon 1441, a mutational hotspot region of the *LRRK2* gene, are rare in Latin America. We only identified three carriers, two with p.R1441G and one with p.R1441C. It is interesting that neither of the p.R1441G carriers reported family history of the disease despite the fact that high penetrance rates have been reported for this mutation; 83.4% by the age of 80 years.^20^ The p.R1441G carrier from Peru presented with clinically typical PD at the age of 29, which is more than two decades lower than the mean age of onset reported for patients with p.R1441G in other cohorts.^22, 23^ In contrast, his 89 year old mother and 54 year old brother both carry the mutation which have not shown signs of parkinsonism on serial neurological examinations, demonstrating that penetrance for this mutation can be highly variable. The clinical features of the proband were characterized by gradual progression over two decades, and good response to levodopa with late onset of motor fluctuations. This is consistent with the more benign phenotype previously reported in other PD patients with p.R1441G.^24, 25^ The reason for the unusually early age at onset of the proband is not clear.^21^ He was negative for both point and dosage mutations in the *PARK2* gene (data not shown), the most frequent genetic cause of early-onset PD, as well as for mutations in *GBA* which not only increase risk for PD but also lower the age at onset by at least 4 years.^26^ It is possible that environmental exposures or other genetic factors not tested might influence the age at onset in this instance. All of the carriers in this family share the common haplotype which is thought to have originated in the Basque region during the VII century. This agrees with the historic influence of Spanish colonizers in the Peruvian population.^27, 28^

The other p.1441G carrier has been previously described elsewhere.^12^ The low p.R1441G frequency in our sample is somewhat surprising due to the large historical influence of Spain in Latin America. However this is consistent with previous smaller studies in which no carriers were found including patients from Brazil^29^ and Chile.^30^ This low frequency might be explained by many factors including the possibility that the first Spanish colonizers came from regions in Spain with a low mutation frequency. Including our carriers, only five have been identified outside of Spain.^10–13^ Regarding the other cases outside of Spain, the Uruguayan and Japanese p.R1441G PD cases showed a novel haplotype suggesting a distinct founder effect,^12, 13^ while for the Mexican and North American cases no haplotype analysis was reported.^10, 11^

We also identified a patient carrying p.R1441C, which we believe is the first report of this mutation in South America. Most of the families previously reported with this mutation are from Europe or the US, except for one from Asia. At least four different haplotypes have been observed in individuals with p.R1441C.^31^ Our patient reported that his paternal relatives originated in Spain, while his maternal relatives came from Lebanon.

The proband of our p.R1441C family was a 63 year old male who presented with bradykinesia and rigidity in the right upper limb at the age of 44 years. He was started on levodopa therapy 3 years later with a clear symptomatic response. He has never displayed a resting tremor, but does have a slight bilateral postural tremor. These clinical characteristics are somewhat atypical because nearly 60% of patients with p.R1441C report resting tremor, as their initial symptom and <20% of carriers have an age at onset of <50 years.^21, 31^ The proband developed motor complications after 3 years of levodopa treatment, and now has a very short duration of drug action (90–120 min) and moderate peak-dose dyskinesias with generalized choreic and dystonic movements. He is in Hoehn and Yahr stage III, when in the “on state”. No significant cognitive impairment was observed on detailed neuropsychological testing at age 59. He reported nine relatives affected with parkinsonism, however DNA was not available for additional individuals of the family (Fig. 2).

Finally, *LRRK2* p.Q1111H was originally reported in two siblings with PD from the U.S., but the pedigree was too small to assess segregation.^32^ However this variant was nominated as potentially pathogenic since it was not found in almost 400 non-Hispanic white controls. We later demonstrated that p.Q1111H is a common variant that is restricted to populations of Amerindian origin.^19^ In an analysis of 1150 PD patients and 310 healthy controls from Peru and Chile we showed a trend toward an association between p.Q1111H and PD (OR 1.38; *p* = 0.10). Here we attempted to validate these findings in the largest Latin American PD cohort ever assembled. However, after adjusting for important covariates we observed no association between p.Q1111H and PD (OR 1.02, *p* = 0.873). This suggests that p.Q1111H is a “benign” population specific SNP.

Human genetics is proving to be a key component of personalized medicine. However, most PD genetic studies have focused on individuals of European origin, and little is known about genetic risk factors and causal genes for PD in other populations. Without such information, it will be difficult to individualize new treatments for PD patients from underrepresented groups, which might further increase existing social disparities. Furthermore, genetic analyses of non-European populations might yield new PD genes that could elucidate novel therapeutic targets to benefit all patients. The data presented here begin to address this gap in knowledge, and we have just initiated large scale studies in the LARGE-PD cohort that will further define the genetic profile of PD in Latinos.

## Materials and methods

### Subjects

We screened a total of 1734 PD patients and 1097 healthy controls recruited in Argentina, Brazil, Colombia, Ecuador, Peru, and Uruguay as part of the Latin American Research Consortium on the Genetics of Parkinson’s disease (LARGE-PD). All patients were evaluated by a movement disorders specialist at each of the sites and met UK PD Society Brain Bank clinical diagnostic criteria. The characteristics of this cohort are presented in Table 1. Data for a subset of these subjects has been previously published in analyses of p.G2019S and codon 1441 mutations (*n* = 365), and for p.Q1111H (*n* = 940).^12, 19^

**Table 2:** Allele and genotype frequencies of *LRRK2* p.Q1111H (rs78365431)

Site	Affection status	Samples No.	Genotype GG No. (%)	Genotype GT No. (%)	Genotype TT No. (%)	G allele No. (%)	T allele No. (%)	Odds ratio (95% CI)	*P*-value
Argentina	Cases	179	175 (97.8)	4 (2.2)	0	354 (98.9)	4 (1.1)	NA	NA
	Controls	NA	NA	NA	NA	NA	NA		
Brazil	Cases	412	408 (99.0)	4 (1)	0	820 (99.5)	4 (0.5)	0.93 (0.24-3.51)	0.919
	Controls	283	281 (99.3)	1 (0.35)	1 (0.35)	563 (99.5)	3 (0.5)		
Colombia	Cases	197	188 (95.4)	9 (4.6)	0	385 (97.7)	9 (2.3)	1.7 (0.56-5.21)	0.342
	Controls	184	179 (97.3)	5 (2.7)	0	363 (98.6)	5 (1.4)		
Ecuador	Cases	85	80 (94.1)	5 (5.9)	0	165 (97.1)	5 (2.9)	NA	NA
	Controls	NA	NA	NA	NA	NA	NA		
Peru	Cases	536	444 (82.8)	82 (15.3)	10 (1.9)	970 (90.5)	102 (9.5)	1.03 (0.72-1.46)	0.884
	Controls	248	204 (82.3)	42 (16.9)	2 (0.8)	450 (90.7)	46 (9.3)		
Uruguay	Cases	280	276 (98.6)	4 (1.4)	0	548 (99.3)	4 (0.7)	0.67 (0.25-1.88)	0.447
	Controls	272	265 (97.4)	7 (2.6)	0	537 (98.7)	7 (1.3)		
Combined	Cases	1689	1571 (93.0)	108 (6.4)	10 (0.6)	3250 (96.2)	128 (3.8)	1.02 (0.75-1.40)	0.873
	Controls	987	929 (94.1)	55 (5.6)	3 (0.3)	1913 (96.9)	61 (3.1)		

Estimated odds ratios (ORs) with confidence intervals (CIs) and *p*-values result from logistic regression models adjusted for age, sex, and site (for the combined sample only)

### Genetics

Genomic DNA was extracted from peripheral blood samples using standard methods. All samples were screened for p.G2019S and p.Q1111H by TaqMan assay and a cluster of four substitutions in codon 1441 (p.R1441C/G/H/S) by sequencing *LRRK2* exon 31 using the Applied Biosystems Big-Dye Terminator v3.1 Cycle Sequencing Kit. Sequence data were analyzed using Mutation Surveyor (SoftGenetics, PA). All p.G2019S carriers were verified by Sanger sequencing using the same methods as for exon 31.

Haplotype analyses for p.R1441G were performed using 5 SNPs and 10 microsatellite markers spanning 6 Mb across the *LRRK2* region^28^ (Supplementary Table 1). The haplotype background for p.G2019S was determined by genotyping rs28903073. The “A” allele for this SNP has a frequency of <0.1% and if observed indicates the presence of the rare haplotype shared by most p.G2019S carriers.^4^

SNP markers were genotyped by Sanger sequencing using previously described methods.^9^ Microsatellites were amplified by PCR using fluorescently labeled Forward primers, run on an ABI PRISM 3130 Genetic Analyzer, and analyzed using GeneMapper 4.0 software (Applied Biosystems, CA).

We also screened a total of 214 individuals from five of the six participating sites (range, 17–50) using a custom panel of 29 ancestry informative markers (AIMs) to estimate the proportion of admixture for the four continental population groups (Asian, African, European, and Amerindian) present in each site (Supplementary Methods and Supplementary Tables 2 and 3). Genotyping was performed using TaqMan assays on the Fluidigm BioMark HD System. We used STRUCTURE (http://pritch.bsd.uchicago.edu/structure.html) to estimate the percent ancestry for our samples with reference to the HDGP + HapMap Phase III groups.

### Statistical analysis

Association of PD with p.Q1111H was assessed by logistic regression analysis under an additive model, adjusting for sex and age. We also included site as a covariate in the analysis of the combined cohort. Analyses were performed using Stata software (version 14.0; StataCorp LP, College Station, TX).

## Electronic supplementary material

Supplement Tables 1-3 and Supplement methods
