# Erratum: Variable frequency of LRRK2 variants in the Latin American research consortium on the genetics of Parkinson’s disease (LARGE-PD), a case of ancestry

**DOI:** 10.1038/s41531-017-0025-1

**Published:** 2018-01-19

**Authors:** Mario Cornejo-Olivas, Luis Torres, Mario R. Velit-Salazar, Miguel Inca-Martinez, Pilar Mazzetti, Carlos Cosentino, Federico Micheli, Claudia Perandones, Elena Dieguez, Victor Raggio, Vitor Tumas, Vanderci Borges, Henrique B. Ferraz, Carlos R. M. Rieder, Artur Shumacher-Schuh, Carlos Velez-Pardo, Marlene Jimenez-Del-Rio, Francisco Lopera, Jorge Chang-Castello, Brennie Andreé-Munoz, Sarah Waldherr, Dora Yearout, Cyrus P. Zabetian, Ignacio F. Mata

**Affiliations:** 1 Neurogenetics Research Center, Instituto Nacional de Ciencias Neurologicas, Lima, Peru; 2 Northern Pacific Global Health Research Training Consortium, Bethesda, MD USA; 3 Movement Disorders Unit, Instituto Nacional de Ciencias Neurologicas, Lima, Peru; 4 Universidad Nacional Mayor de San Marcos, Lima, Peru; 5 Universidad Peruana Cayetano Heredia, Lima, Peru; 6 Hospital de Clínicas José de San Martín, Universidad de Buenos Aires, Buenos Aires, Argentina; 7 Neurology Institute, Universidad de la Republica, Montevideo, Uruguay; 8 Department of Genetics, Facultad de Medicina, Universidad de la Republica, Montevideo, Uruguay; 9 Ribeirão Preto Medical School, Universidade de São Paulo, Ribeirão Preto, Brazil; 10 Movement Disorders Unit, Department of Neurology and Neurosurgery, Universidade Federal de São Paulo, São Paulo, SP Brazil; 11 Hospital de Clínicas de Porto Alegre, Porto Alegre, Brazil; 12 Neruroscience Research Group, Medical Research Institute, Universidad de Antioquia, Medellin, Colombia; 13 Department of Genetics, School of Medicine, Universidad de Guayaquil, Hospital Luis Vernaza, Guayaquil, Ecuador; 14 Service of Neurology, Hospital Luis Vernaza, Guayaquil, Ecuador; 15 Veterans Affairs Puget Sound Health Care System, University of Washington, Seattle, WA USA; 16 Department of Neurology, University of Washington, Seattle, WA USA

**Erratum to:** *npj Parkinson’s Disease* (2017); doi:10.1038/s41531-017-0020-6; Published online 02 June 2017

The original version of this article contained an error in the name of gene *LRRK2*, which was incorrectly given as *Leucine Repeat Rich Kinase 2*. The correct name is *Leucine Rich Repeat Kinase 2*. This has now been corrected in the HTML and PDF versions of this Article.

