# Supplementary material for: Variable frequency of LRRK2 variants in the Latin American research consortium on the genetics of Parkinson’s disease (LARGE-PD), a case of ancestry
Source: NPJ Parkinsons Dis. 2017 Jun 2;3:19. doi: 10.1038/s41531-017-0020-6 (PMC5460260; doi:10.1038/s41531-017-0020-6)
Supplement: Supplementary file 1 — Supplement Tables 1-3 and Supplement methods [file 41531_2017_20_MOESM1_ESM.docx]

**Supplementary e-table 1. Comparison of R1441G-containing haplotypes across the *LRRK2* region**

|  |  |  | Proband | Son | Son | Father | Mother | Brother |
| --- | --- | --- | --- | --- | --- | --- | --- | --- |
| **Location** | **Marker** | **Shared^*^** | **II-2** | **III-1** | **III-2** | **I-1** | **I-2** | **II-3** |
| 33305760 | **D12S2080** | **188** | **188**/192 | **188**/188 | **188**/192 | 192/192 | **188**/1192 | **188**/192 |
| 34142287 | **rs1511547** | **T** | **T/C** | **T/**T | **T**/C | C/C | **T/**C | **T/**C |
| 34142413 | **rs55917927** | **C** | T/**C** | T/**C** | T/T | T/T | T/**C** | T/**C** |
| 34142467 | **rs56260627** | **A** | **A/**G | **A/**G | G/G | G/G | **A/**G | **A/**G |
| 37708307 | **rs10876410** | **A** | **A**/A | **A**/A | **A**/A | **A**/A | **A**/A | **A**/A |
| 38738007 | **D12S2194** | **249** | **249**/249 | **249**/249 | **249**/249 | **249**/249 | **249**/253 | **249**/249 |
| ***LRRK2*** | **D12S2516** | **252** | **252**/254 | **252**/254 | 254/254 | 254/254 | **252**/254 | **252**/254 |
|  | **R1441G** | **G** | **G/**C | **G/**C | C/C | C/C | **G/**C | **G/**C |
|  | **M1646T** | **C** | T/**C** | T/**C** | T/T | T/T | T/**C** | T/**C** |
|  | **D12S2518** | **154** | **154**/156 | **154**/156 | **154**/170 | **154**/156 | **154**/156 | **154**/156 |
| 39116885 | **D12S2519** | **138** | **138**/140 | 132/**138** | 132/140 | 132/140 | 134/**138** | **138**/140 |
| 39120098 | **D12S2520** | **248** | **248**/260 | **248**/251 | 251/260 | 257/260 | **248**/257 | **248**/260 |
| 39128754 | **D12S2521** | **323** | 319/**323** | **323**/367 | 319/367 | 319/359 | **323**/371 | 319/**323** |
| 39132380 | **D12S2522** | **281** | **281**/285 | **281**/299 | 285/299 | 285/297 | **281**/297 | **281**/285 |
| 39176380 | **D12S2517** | **184** | **184**/206 | **184**/190 | 190/206 | 182/206 | **184**/188 | **184**/206 |
| 39312730 | **D12S1048** | **211** | **211**/211 | **211**/226 | **211**/226 | **211**/211 | **211**/220 | **211**/211 |

^*^ indicates the haplotype shared among patients with R1441G from Northern Spain ^1^. Allele sharing is indicated in bold.

1 Mata, I. F. *et al.* Lrrk2 R1441G-related Parkinson's disease: evidence of a common founding event in the seventh century in Northern Spain. *Neurogenetics* **10**, 347-353, doi:10.1007/s10048-009-0187-z (2009).

**Supplementary methods: Ancestry informative markers (AIMs)**

Population Controls and Ancestry Samples

We used the Human Genome Diversity Panel (HGDP) data set, filtered to include only individuals from populations in Africa, Europe, East Asia, and America (Biswas et al. 2009). This totaled non related 553 individuals, which included 102 from Africa, 158 from Europe, 229 from East Asia, and 64 from the Americas.

Identification and Selection of the AIMs

Based on the HGDP data set, we performed a principle component analysis (PCA) on 643,884 sites. We then correlated each SNP with the first three principle components (PC) using Spearman’s rho, which does not assume normality. Next, we selected the top 200 most correlated SNP’s for each PC and found pairwise r2 between all combinations, even across chromosomes. This allowed us to select which SNP’s were correlated to the PC for different reasons (ie not also correlated with each other). With these two pieces of information (r2 and PC rho) we performed the following algorithm: 1) selected the best SNP for each PC axis based on the absolute value of rho, 2) removed any other SNP in the list with an r2 with the selected SNP greater than 0.5, and 3) repeated the process to select 10 SNPs for each axis. This created an LD aware set of 30 SNP’s with sufficiently distinct properties of correlation. One of the SNPs failed to work in the TaqMan assay, but we proceeded without it giving us a total of 29 SNPs that work effectively and efficiently (Supplementary e-table 2).

Validation

We validated these SNPs on an independent data set using the HapMap Phase III data (http://hapmap.ncbi.nlm.nih.gov/) and selecting 27 of the 29 identified SNPs (2 are missing in this data set and are designated in Table 1). We ran PCA for these SNPs on all populations, except Gujarati Indians in Houston TX. This included a) Europeans (descendants of northern and western European decent [CEU], and Italians [TSI]), b) East Asians (Japanese [JPT]; Han Chinese in Beijing, China [CHB]; and Chinese in Denver, CO [CHD]), and c) Africans (Eastern: Luhya in Webuye, Kenya [LWK] and Maasai in Kinyawa, Kenya [MKK]; Western: Yoruba in Ibadan, Nigeria [YRI]). Amerindian populations were unavailable at the time. In addition two admixed populations were also included (African Americans from the Southwest USA [ASW]; and Mexicans in Los Angeles, CA [MEX]).

LARGE-PD analysis

We randomly sampled 214 individuals from the LARGE PD cohort as follows: 39 Peruvian mestizos; 40 Peruvian Ameridian; 17 Uruguayan; 21 Argentinian, 47 Colombian and 50 Brazilian. Based on HapMap III and HGDP, we constructed a combined reference panel consisting on European, Africans, Amerindians and East Asians for a total of 1354 individuals. We used STRUCTURE (http://pritch.bsd.uchicago.edu/structure.html) to estimate the percent ancestry for our samples with reference to the HDGP+HapMap Phase III groups. Thus, we assumed k=4 ancestral groups, 10,000 burn-in iterations and 20000 MCMC iterations and default settings. We made 4 different runs and then used CLUMPP to align these runs and took the average of the 4 runs sorted by percentage of European ancestry (Supplementary e-table 3).

**Supplementary e-table 2. List of SNPs selected for our AIMs panel**

| **RS id** | **Chromosome** | **Position**  **(Build 36.3)** |
| --- | --- | --- |
| rs7541084 | 1 | 51,762,179 |
| rs8179271* | 1 | 153,803,362 |
| rs1834619 | 2 | 17,764,966 |
| rs1348587 | 2 | 154,440,039 |
| rs1347886 | 2 | 164,854,620 |
| rs17627058 | 2 | 177,478,841 |
| rs9840466 | 3 | 72,175,958 |
| rs842223 | 3 | 196,968,747 |
| rs10008281 | 4 | 100,361,325 |
| rs1010574 | 5 | 10,847,152 |
| rs149138 | 5 | 55,562,970 |
| rs10434525 | 5 | 59,592,218 |
| rs10079352 | 5 | 117,522,539 |
| rs1366220 | 5 | 153,477,973 |
| rs3997520 | 6 | 44,659,465 |
| rs10763013 | 10 | 55,283,129 |
| rs2716454 | 11 | 24,710,115 |
| rs590616 | 11 | 100,353,685 |
| rs932055** | 12 | 22,591,655 |
| rs1924373 | 13 | 49,843,707 |
| rs9568431 | 13 | 49,953,648 |
| rs10483393 | 14 | 31,530,235 |
| rs3211166 | 14 | 68,772,911 |
| rs2676765 | 15 | 54,636,697 |
| rs17675813 | 16 | 64,367,041 |
| rs4924980 | 17 | 19,145,456 |
| rs9960403 | 18 | 13,427,993 |
| rs6094461* | 20 | 44,823,556 |
| rs433632 | 21 | 42,893,492 |
| rs131026 | 22 | 47,561,914 |

* SNPs not included in the HapMap analysis; ** Closest SNP to *LRRK2*

**Supplementary e-table 3. Summary of ancestry estimates for each of the 4 continental groups in each of the HapMap3 and LARGE-PD populations.**

| **POP** | **STUDY** | **African** | **European** | **East_Asian** | **Amerindians** | **N** |
| --- | --- | --- | --- | --- | --- | --- |
| **Africa** | HapMap3 + HGDP | 0.931 | 0.066 | 0.002 | 0.001 | 448 |
| **America** | HapMap3 + HGDP | 0.012 | 0.019 | 0.006 | 0.963 | 64 |
| **East_Asia** | HapMap3 + HGDP | 0.004 | 0.023 | 0.969 | 0.004 | 484 |
| **Europe** | HapMap3 + HGDP | 0.02 | 0.963 | 0.009 | 0.009 | 358 |
| **MEX** | HapMap3 + HGDP | 0.06 | 0.518 | 0.013 | 0.409 | 50 |
| **Argentina** | LARGE-PD | 0.027 | 0.841 | 0.005 | 0.128 | 21 |
| **Peru (Amerindians)** | LARGE-PD | 0.006 | 0.089 | 0.004 | 0.901 | 40 |
| **Peru** | LARGE-PD | 0.061 | 0.253 | 0.004 | 0.681 | 39 |
| **Uruguay** | LARGE-PD | 0.015 | 0.827 | 0.048 | 0.109 | 17 |
| **Colombia** | LARGE-PD | 0.126 | 0.647 | 0.003 | 0.224 | 47 |
| **Brazil** | LARGE-PD | 0.168 | 0.729 | 0.025 | 0.077 | 50 |
